# A Microscale Linear Phased-Array Ultrasonic Transducer Based on PZT Ceramics

**DOI:** 10.3390/s19051244

**Published:** 2019-03-12

**Authors:** Xue-Jiao Jiang, Meng-Wei Liu, Fang-Fang Shi, Wen Wang, Xian-Mei Wu, Jia-Yi Chen

**Affiliations:** 1 Institute of Acoustics, Chinese Academy of Sciences, Beijing 100190, China; jiangxuejiao16@mails.ucas.ac.cn (X.-J.J.); fangfangshi@mail.ioa.ac.cn (F.-F.S.); wangwenwq@mail.ioa.ac.cn (W.W.); wuxm@mail.ioa.ac.cn (X.-M.W.); chenjiayi@mail.ioa.ac.cn (J.-Y.C.); 2 School of Electronic, Electrical and Communication Engineering, University of Chinese Academy of Sciences, Beijing 100190, China

**Keywords:** microscale, PZT, traditional planar ultrasonic phased array, the micro processing technology, beam forming, echo imaging

## Abstract

In this paper, a microscale high-frequency ultrasonic transducer was prepared by combining traditional planar ultrasonic phased-array technology and micro processing technology. The piezoelectric ceramic material PZT was used as the functional material of the transducer. The number of the arrays was 72, the width of each array was 50 μm, the pitch of each array was 70 μm, and the length of each array was 3 mm. The PZT chip was finely ground to a thickness of 130 μm and could reach a frequency of 10 MHz. The experimental platform of micron-scale precision was set up for a beam-forming lateral sound field test and imaging experiment to validate the theoretical analysis. The echo imaging test showed that a mold with a feature size of about 400 μm could be imaged well.

## 1. Introduction

Ultrasonic transducers have been used in many applications and are famous for nondestructive testing (NDT) and medical imaging. Ultrasonic imaging as a means of detection, compared with other methods, has good directionality, strong penetrating ability, the ability to obtain more concentrated sound energy, and has long-distance propagation in water. Conventional ultrasonic transducers are largely based on bulk piezoelectric ceramics. In contrast, microscale devices hold great promise for medical applications. These devices also help achieve a compact form factor, higher resolution in imaging and therapy, and higher sensitivity [1,2]. The linear array scanning B-scan ultrasonography system currently used for medical detection is generally bulky and cannot meet the detection imaging of micro-scale biometrics. At present, the micro-transducers used for micro-structure detection are mainly micro-electromechanical ultrasonic transducers (MUTs) with a compliant membrane structure [3,4], including capacitive micromachined ultrasonic transducers (cMUTs) and piezoelectric micromachined ultrasonic transducers (pMUTs) [5,6]. MUTs mainly use silicon-based ultrasonic transducer array technology prepared by MEMS technology [7], and their working mode bends the vibrations of the piezoelectric transducer. They usually need silicon and a micro-machining process such as bonding. Due to deep silicon etching and the limitations of the silicon process, the complexity of this preparation method is greatly exacerbated. cMUTs have an almost perfect theoretical coupling [8], there are practical operational and fabrication considerations that limit the achievable coupling. Another practical issue with cMUTs is the need to have different designs for sending and receiving elements [9]. The coupling coefficient and other key indicators of pMUTs are significantly affected by the silicon membrane [10], piezoelectrical materials, and top electrode thickness as well as the top electrode design [11]. This greatly increase the difficulty of and the cost of the preparation process. 

In view of the above disadvantages in size, performance, and preparation process, a micro scale high-frequency ultrasonic transducer is presented in this paper and was prepared by combining the traditional planar ultrasonic phased-array technology and the micro processing technology. Linear arrays with electronic scanning started replacing fixed-focus mechanical sector scanners in the 1970s, providing greatly improved resolution and faster image formation [12]. Compared with the MEMS process, they are mature and greatly reduce the difficulty and cost of the fabrication process. The piezoelectric ceramic material PZT, a material with high piezoelectric coefficients and high relative permittivity [13], is used as the functional material of the transducer. The micro-electrode arrays with transverse dimensions of micron scale are prepared by microfabrication, and the thickness of the transducer can reach in the order of 10 to 100 microns by fine grinding. With high detection resolution, large detection depth, and relatively simple preparation technology, they can be used for ultrasonic detection of micro-scale characteristic structures, such as in skin testing.

## 2. Sound Field Analysis of the Microscale Transducer

The sound field characteristics of microscale phased-array transducers are the determinants of whether the echo information in the detected region can be obtained and effectively utilized to achieve high resolution and large detection depth, and they are also the main basis for the optimization design of transducers [14]. The pressure field in fluid medium produced by a rectangular array element can be calculated by use of the well-known Rayleigh–Sommerfeld integral [15], given by Equation (1).

(1) $$p(x,\omega )\approx -\frac{i\omega \rho u}{2\pi }\int_{s}\frac{\exp(ikr_{0})}{r_{0}}ds$$

where, ω is the angular frequency, ρ is the density, u is the velocity on the transducer face S, $r_{0}$ is the distance from the field point in space to the point source, where each point on the array element can be regarded as the point source, and k is the wave number.

**Figure 1:**
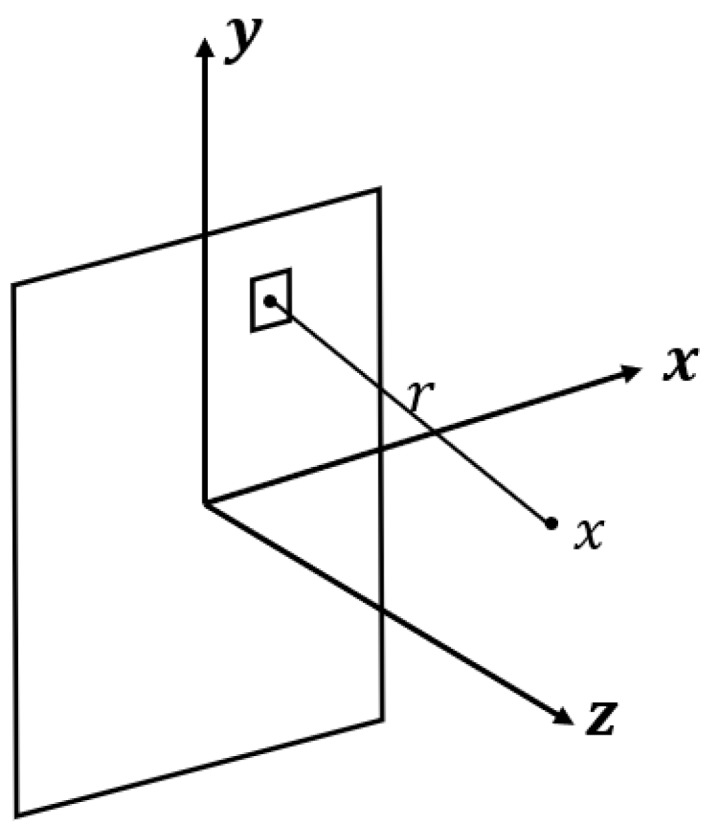
Fast algorithm coordinate system.

It is complicated and slow to calculate the pressure field directly according to Equation (1). We used the fast algorithm proposed by Ocheltree et al. [16] for numerical calculation, and its coordinate system is shown in Figure 1. The rectangular array element is divided into a series of rectangular micro-elements that are large enough to be effective but small enough relative to the point source to greatly simplify the integral calculation by using the fast algorithm. After the rectangular matrix element is divided, its sound pressure is distributed as the sum of the sound pressure superposition of each rectangular sub-matrix element, as shown in Equation (2).

(2) $$p(x,\omega )\approx -\frac{i\omega \rho }{2\pi }\sum_{n=1}^{N}u_{n}\frac{\exp(ikr)}{r}\Delta s$$

where, ω is the angular frequency, ρ is the density, r is the distance from the field point x in space to the center of a sub-matrix element, k is the wave number, S is the area of a rectangular matrix, N is the number of the array elements, $u_{n}$ is the vibration velocity of the n^th^ rectangular sub-matrix element, and all the sub-matrix elements have the same vibration velocity for the rectangular matrix element under the same excitation.

The linear phased array is composed of several independent rectangular array elements combined according to certain shapes and sizes. Each array element has its own independent transmitting and receiving circuit, it is excited in sequence according to a certain delay law, and the ultrasonic emission beam that is generated is synthesized in the detection space to form the focus and focusing direction [17]. By changing the delay rules for excitation of each array element, the focus position and its transverse sound field can be changed to form a scanning focus within a certain space range. The precise delay design directly affects the focusing effect, and also relates to the detection performance and imaging resolution. Based on Huygens principle, the radiation sound field of the array can be superimposed by the radiation sound field of each array element with an appropriate delay [18], given by Equation (3).

(3) $$P=\sum_{m=1}^{N_{m}}p(x,\omega )e^{j\omega t_{d}\left(m\right)}$$

where, the delay function formula of the *m*^th^ matrix element is

(4) $$t_{d}(m)=\frac{R(m)}{c}\;m=1,2\ldots \ldots N_{m}$$

where, $N_{m}$ is the number of array elements, *c* is sound velocity, and R(m) is the distance from the center of the *m*^th^ array element to the focus point.

**Figure 2:**
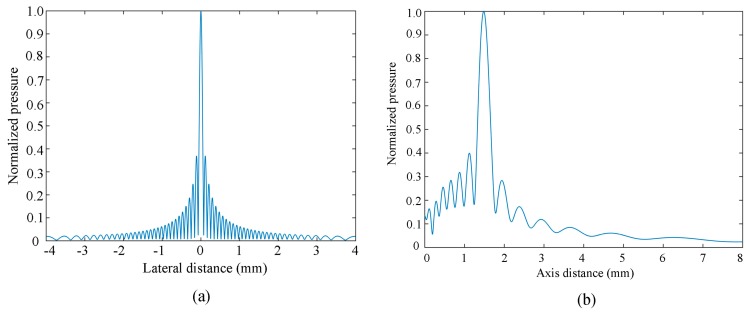
The lateral sound field (**a**) and axis sound field distribution (**b**).

Using Equation (3), the number of linear array elements is 72, the array element width is 50 μm, the array element height is 0.5 mm, the center frequency is 10 MHz, the array element center spacing is 70 μm, and the sound velocity is 1.5 mm/μs. The focus position is F(0, 0, 1.5 mm). The lateral sound field and axis sound field distribution are shown in Figure 2. The longitudinal sound field reaches its maximum at the focus, as shown in Figure 2b. For the lateral sound field, the main lobe, side lobe, and grating lobe are the main factors affecting it. An unsatisfactory sound field can cause resolution artifacts, grating-valve effect artifacts, and side-lobe effect artifacts in ultrasound imaging [19]. Next, we discuss the influence of array element parameters on the lateral sound field.

**Figure 3:**
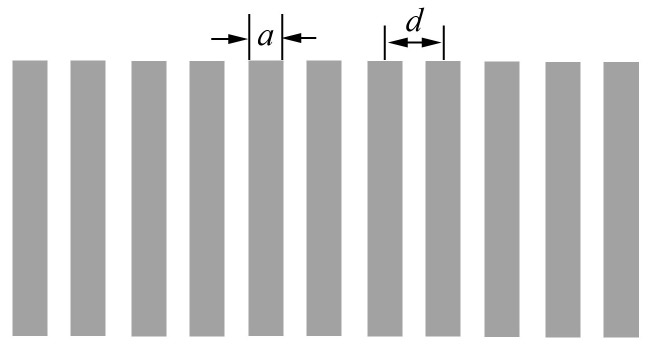
One-dimensional rectangular array.

According to Equation (3), the number of array elements $N_{m}$, the width of array elements a, and the pitch of array elements d (as shown in Figure 3) directly affect the directional sound field of microscale phased-array ultrasonic transducers, which is an important parameter for the design optimization of phased-array ultrasonic transducers. The transverse sound field of microscale linear phased-array ultrasonic transducers should have narrower main lobe width, higher main lobe strength, lower side lobe and no gating lobe. Next, the influence of parameter changes of microscale linear phased-array elements on transverse sound fields of the transducer are discussed, and theoretical analysis is carried out to provide theoretical reference for preparation of the transducer.

### 2.1. Influence of Array Parameters on the −3 dB Main Lobe Width and Theoretical Analysis

The imaging resolution of a microscale transducer is closely related to the main lobe width in a transverse sound field distribution. The narrower the main lobe width is, the higher is the imaging resolution is. In this paper, −3 dB main lobe width is taken as the reference, and a −3 dB main lobe width is the distance between two points of the corresponding transverse distance axis when the normalized sound pressure is −3 dB. If the main lobe is not ideal, resolution artifacts are likely to occur. Due to the limitation of the spatial resolution of the system, any object with a distance less than the resolution of two or more points can only be displayed as a larger object in the image, which is often referred to as low horizontal or vertical resolution [19].

**Figure 4:**
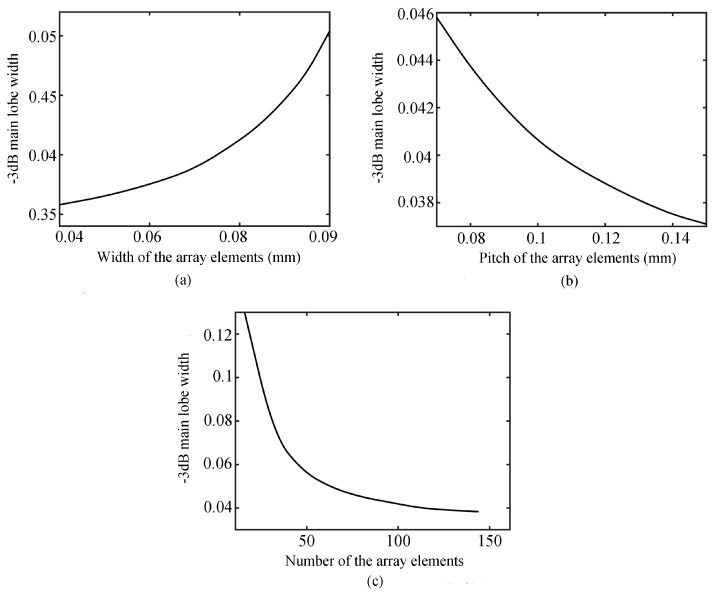
Relationship curve of array width (**a**), pitch (**b**), and number (**c**) with the transverse sound field of the −3 dB main lobe width.

When the focus position is F(0, 0, 1.5 mm), the pitch is 160 μm, and the number of array elements is 72, Figure 4a shows that the −3 dB main lobe width tends to increase with an increase in the width of the array element when the operating frequency is 15 MHz. In addition, when the operating frequency is 15MHz, the focus position is F(0, 0, 1.5 mm), the width is 50 μm, and number of array elements is 72, the −3 dB main lobe width tends to decrease as the pitch of the array element increases, as shown in Figure 4b. Figure 4c shows that when the operating frequency is 15 MHz, the focus position is F(0, 0, 1.5 mm), the width is 50 μm, and the pitch of array elements is 70 μm, the −3 dB main lobe width tends to decrease as the number of the array elements increases. On the whole, increasing the number and pitch of the array elements, or reducing the width of the array element, can reduce the width of the −3 dB main lobe to improve the transverse resolution. However, increasing the number of array elements will not only increase the preparation cost of the transducer, but also increase the overall size of the transducer, which is not conducive to the detection and imaging of microscale structure characteristics. Increasing the pitch of array elements will increase the overall size of the transducer, which does not meet the size requirement of microscale transducers. Besides, the reduction of array width is limited by the fabrication process of piezoelectric ceramic wafers, and the fabrication cost is increased. Therefore, in the optimization analysis of horizontal resolution, all the factors need to be balanced; instead of blindly increasing or decreasing a certain parameter, an optimal solution should be found according to the actual situation.

### 2.2. Influence of Array Parameters on the Intensity of the Main Lobe and Theoretical Analysis

The main lobe intensity in the transverse sound field is the key factor affecting the detection depth range of the transducer. The larger the main lobe intensity is, the larger is the detection depth range. In actual ultrasonic detection and imaging experiments, it is sometimes necessary to detect the 3D features of the subsurface as well as the structural surface of the micro-size features. Therefore, a large detection depth range is one of the important performance indicators of transducers.

**Figure 5:**
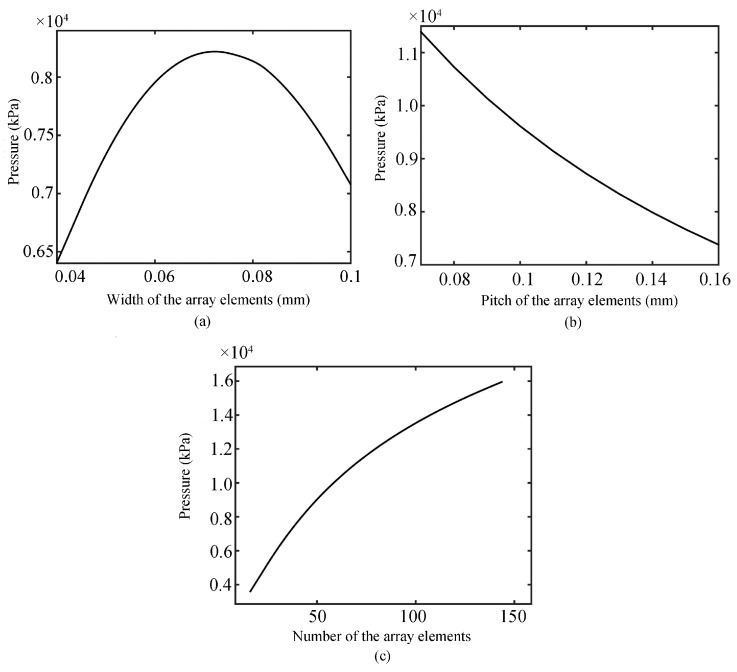
Relationship curve of array width (**a**), pitch (**b**), and number (**c**) with the transverse sound pressure.

When the focus position is F(0, 0, 1.5 mm), the pitch is 160 μm, and the number of array elements is 72, Figure 5a shows that the intensity of sound pressure increases first and then decreases with the increase of array element width when the operating frequency is 15MHz. When the operating frequency is 15 MHz, the focus position is F(0, 0, 1.5 mm), the width is 50 μm, and number of array elements is 72, the intensity of sound pressure tends to increase as the pitch of elements decreases, as shown in Figure 5b. When the operating frequency is 15 MHz, the focus position is F(0, 0, 1.5 mm), the width is 50 μm, and the pitch of array elements is 70 μm, the intensity of sound pressure tends to increase as the number of elements increases, as seen in Figure 5c. In general, as the width of the element is increased within a certain range, it can increase the intensity of the sound pressure; the specific range is affected by the number and pitch of the array elements, which need to be analyzed on a case-by-case basis. However, although this method can increase the intensity of sound pressure, it is not conducive to the main lobe. Reducing the width of the array element can increase the intensity of the sound pressure, but this method is not only limited by the fabrication process of piezoelectric ceramic wafers and the production cost of transducers, but also increases the width of the −3 dB main lobe and reduces the transverse resolution. Increasing the number of array elements can obtain a higher sound pressure intensity, but it will also lead to a larger overall size of the transducer and increased production costs, and it is not conducive to the detection of micro-scale features. Therefore, the preparation of the transducer still needs to consider the balance of each parameter and the actual operation to find the optimal design scheme.

### 2.3. Influence of Array Parameters on the Primary Side Lobe and Theoretical Analysis

Side lobe refers to the wavelet lobe near the main lobe in the transverse sound field distribution. The amplitude of the first side lobe on the left and right sides of the main lobe is the largest, which is called the first side lobe. Side lobes make phased-array sound beams produce energy “leakage” outside the scanning direction, which leads to unsatisfactory sound field distribution of phased-array ultrasonic transducers [20]. The pitch, width, and number of array elements are all important parameters affecting the side lobe. The discussion of the variation law of array parameters and primary side lobes can provide a theoretical basis for the optimal design of the transducer. Strict mathematical formula derivation is very complex; this paper discusses the influence of array parameters on the primary side lobe by MATLAB simulation.

**Figure 6:**
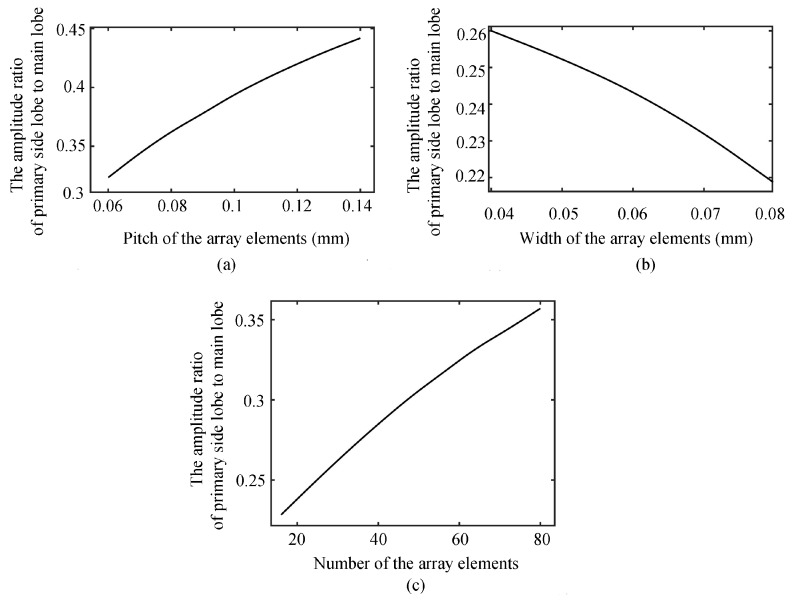
The influence of array parameters, including pitch (**a**), width (**b**), and number (**c**), on the primary side lobe.

When the number of linear array elements is 72, the focus point position is F(0, 0, 1.5mm), the width of the array element is 50 μm, the center frequency is 15 MHz, and the sound velocity is 1.5 mm/μm, Figure 6a shows the relation curve between the ratio of the primary side lobe to the primary lobe and the pitch of array elements. It can be seen that the ratio of the primary side lobe to the main lobe decreases with decreasing array pitch. Figure 6b shows the relationship curve between the ratio of the primary lobe to the main lobe and the width of array elements when the number of array elements is 72, the focus position is F(0, 0, 1.5 mm), the pitch of array elements is 100 μm, the center frequency is 15 MHz, and the sound velocity is 1.5 mm/μm. It shows that as the width of the array increases, the side lobe is suppressed. When the pitch of the linear array elements is 70 μm, the focus position is F(0, 0, 1.5 mm), the width of the array element is 50 μm, the center frequency is 15 MHz, and the sound velocity is 1.5 mm/μm, Figure 6c shows that the size of the side lobe decreases with a decreasing number of array elements. The side lobe cannot be eliminated, only suppressed. According to the above discussion and analysis, increasing the width of array elements, decreasing the pitch of array elements, or decreasing the number of array elements can reduce the ratio of the primary side lobe to the main lobe and make the transverse sound field have a lower side lobe. However, increasing the width of array elements and decreasing the pitch of array elements will lead to an increase of the main lobe width of 3 dB, leading to a decrease of the resolution. In addition, reducing the number of array elements will also have an adverse effect on the width and strength of the main lobe. In the optimal design of side lobe suppression, balancing the advantages and disadvantages is the key to the preparation of the transducer.

### 2.4. Influence of Array Parameters on the Grating Lobe and Theoretical Analysis

The gate lobe is a wave lobe whose amplitude is lower than the main lobe and higher than the side lobe [20]. The presence of a gating lobe not only makes the energy of sound energy “leak”, but also produces a pseudo-signal that interferes with the detection of structural defects. Therefore, the gating lobe must be completely eliminated as it is not conducive to microscale linear phased-array ultrasonic imaging detection.

**Figure 7:**
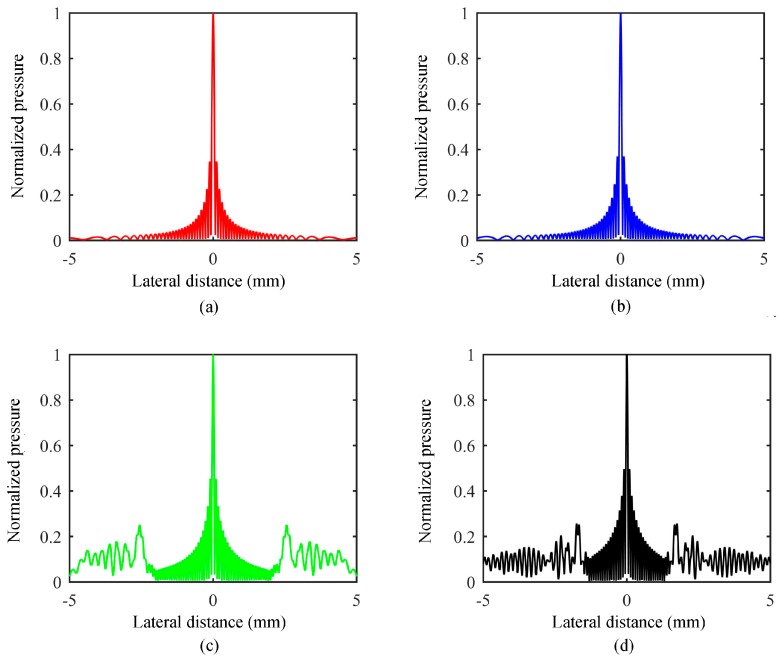
The lateral sound field of the phased array with different element pitches of 60 μm (**a**), 70 μm (**b**), 120 μm (**c**), and 160 μm (**d**).

When the number of linear array elements is 72, the focus position is F(0, 0, 1.5 mm), the width of the array element is 50 μm, the center frequency is 10 MHz, the sound velocity is 1.5 mm/μm, and the wavelength is 150 μm, Figure 7 shows in turn the lateral sound field distribution of microscale linear phased-array with different element pitches of 60 μm, 70 μm, 120 μm, and 160 μm. As shown in Figure 7, when the array element pitch is less than half of the wavelength, the lateral acoustic field did not produce a gating lobe; when the array element pitch increased to more than one-half of the wavelength, the lateral distribution of the sound field produced a gate valve. Therefore, to eliminate the gating lobe in the design of microscale linear phased-array ultrasonic transducers, the array element pitch needs to be smaller than one-half of the wavelength. 

## 3. Structure Design and Fabrication Process

**Figure 8:**
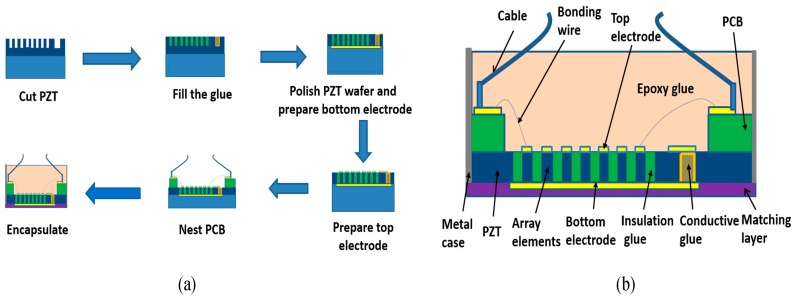
Schematic diagram of fabrication process (**a**) and final prepared transducer (**b**).

**Figure 9:**
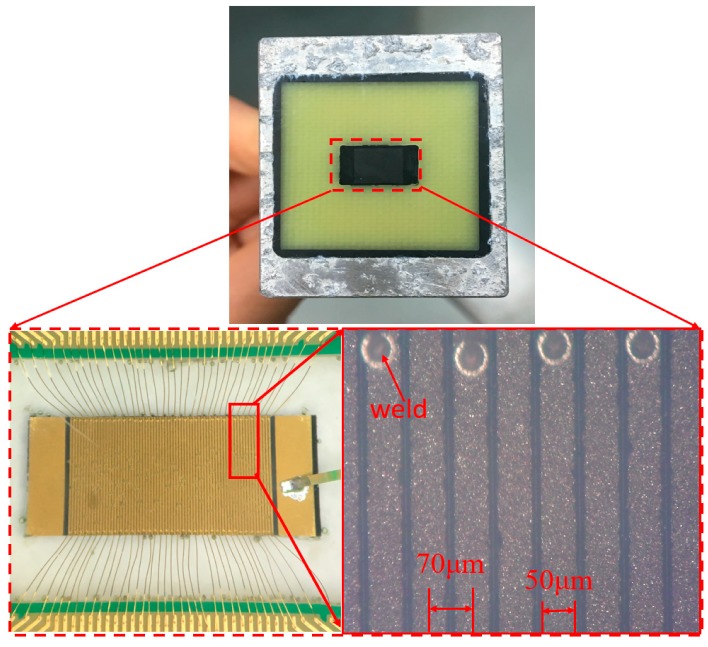
Fabricated transducer.

Combining theoretical analysis with a practical situation, we set the number of array elements to 72, the array element width to 50 μm, and the array element spacing to 70 μm. A 50 μm array element width and 72 array elements ensure that the lateral sound field has a narrow main lobe, which ensures the lateral resolution and the number of array elements is not excessive, reducing the overall size. A 70 μm array spacing ensures high lateral amplitude, suppresses side lobes, and ensures that no grating lobes will appear. The specific preparation process of the transducer designed in this paper is shown in Figure 8a. First, the PZT piezoelectric ceramic substrate was bonded to the support, which was a glass substrate. Then, the PZT substrate was cut by a grinding wheel to form 72 linear transducer elements with the same width of 50 μm and equal pitch of 70 μm. In the second step, one side of the transducer array was cut to obtain a coplanar electrical connection groove on the bottom electrode. The width of the coplanar electrical connection groove of the bottom electrode was 20–30 μm, and the depth was equal to the cutting depth of the transducer array. In the third step, the isolation glue was poured between the transducer elements to reduce the coupling crosstalk when the elements are working. In order to facilitate the subsequent extraction of the bottom electrode, conductive adhesive was poured into the coplanar electrical connection groove of the bottom electrode. The surface of the piezoelectric ceramic substrate was ground and polished to form a smooth surface that was used for semiconductor photolithography. Then, the forth step was to fabricate the bottom electrode and ensure that the bottom electrode was connected to the conductive glue in the coplanar connection groove. The bottom electrode adopted the coating technology of evaporation or sputtering, the material was Au, and the conductivity was good. Next, the fifth step was to remove the bottom support of the piezoelectric ceramic substrate, turn the piezoelectric ceramic substrate over, bond its bottom electrode surface with the support, grind and polish the piezoelectric ceramic surface relative to the bottom electrode, and reduce the thickness of the piezoelectric ceramic to 130 μm. After that, the sixth step was to form the top electrode of the linear array micro transducer, by using semiconductor lithography, etching, or stripping technology, and then prepare the top electrode connected with the conductive adhesive in the coplanar electrical groove of the bottom electrode to facilitate the extraction of the bottom electrode in the later packaging process. The material of the top electrode was Au. Then, the frame-shaped PCB was bonded to the piezoelectric ceramic substrate, and the top electrode on the piezoelectric ceramic was connected to the PCB plate by a wire-bonding process. Finally, the matching layer was prepared, and epoxy resin was poured into it as the backing material. Then, the required PZT-based microscale ultrasonic linear phased-array transducer was encapsulated, as shown in Figure 8b. Optical images of the PZT-based microscale ultrasonic linear phased-array transducer are shown in Figure 9.

## 4. Experimental Testing and Discussion

### 4.1. Transverse Sound Field Test Experiment and Discussion

**Figure 10:**
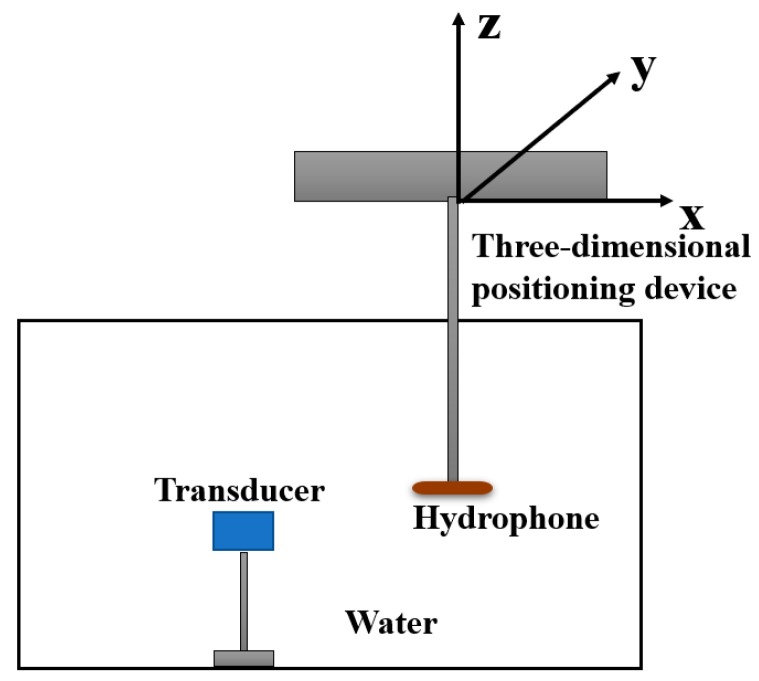
Acoustic pressure measurement setup using a hydrophone.

**Figure 11:**
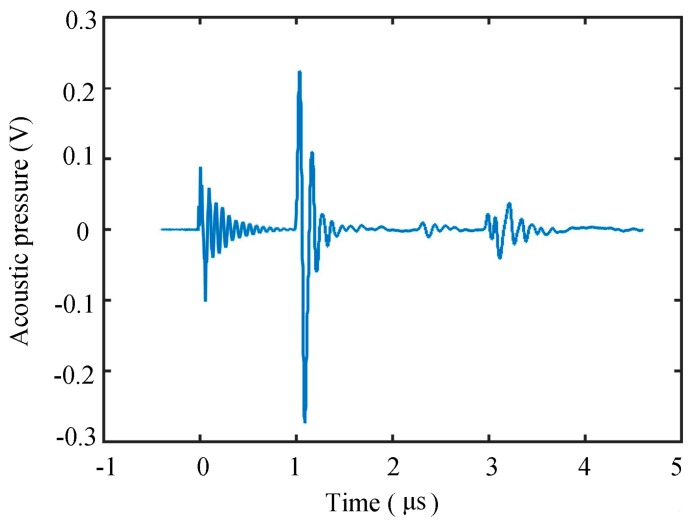
Measured acoustic pressure for a 32-element array with a 70 μm beam-forming pitch.

Transverse sound field test experiments were conducted with the microscale transducer immersed in water. Figure 10 is a diagram of the setup for pressure measurements in water. The micro-transducer was fixed to the fixture, then a needle hydrophone with 1 mm effective diameter was fixed to a three-dimensional positioning device, which was controlled by a motor, and had a moving accuracy of up to one micron. The micro-transducer was driven with pulses of 100 V, 50 ns pulse width, and 1000 Hz in a sequence according to a certain delay law by using a Phascan portable ultrasonic phased-array detector. The selected phase delay of each column was calculated based on the acoustic propagation path length from each array element to the desired focal point. Figure 11 shows the hydrophone measured sound pressure curve at the focus point when the number of linear phased-array elements was 32, the width of the array element was 50 μm, the pitch of array element was 70 μm, the height of array element was 3 mm, the sound velocity was 1500 m/s, and the focus position was (0, 0, 1.5 mm). The value of peak-to-peak (Vpp) of the curve is regarded as the sound pressure at the focus point. 

**Figure 12:**
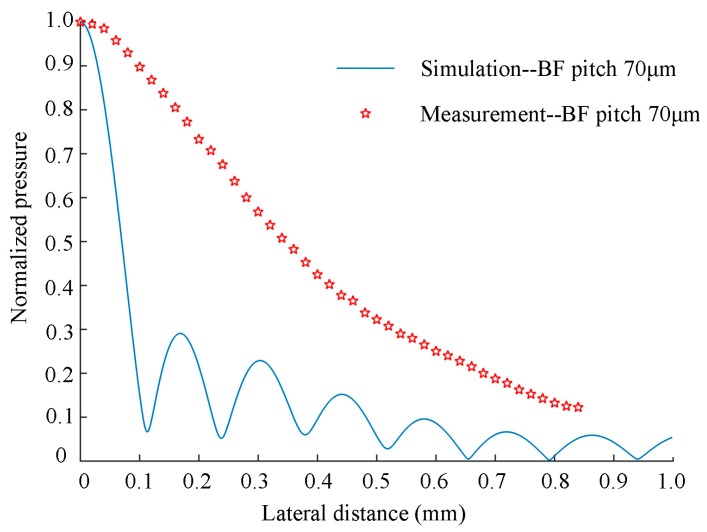
Measured acoustic beam-pattern compared with simulation results for a 32-element array with a 70 μm beam-forming pitch.

After the sound pressure at the focus point was measured, we moved the hydrophone laterally and took a Vpp value reading every 20 μm, and then we generated the specific transverse sound field distribution as shown in Figure 12. Figure 12 shows the theoretical curve and the measured scatter plot. It can be seen that although the general trend is the same, there is a great difference between simulation and experimental results; this difference may be caused by the time-delay resolution of the pulse generator or non-ideal measurement positioning. Another possible cause of the difference is that the simulation uses a continuous-wave (CW) model, while measurements were obtained with a pulsed-wave driving signal. This also explains why the side lobes shown in the simulation results were not observed in the measurement results. In addition, it is also possible that the 1 mm diameter of the hydrophone affected the results as it far exceeded the moving accuracy of 20 microns. Later, we will improve the size of the hydrophone. 

### 4.2. The Echo Imaging Test and Discussion

**Figure 13:**
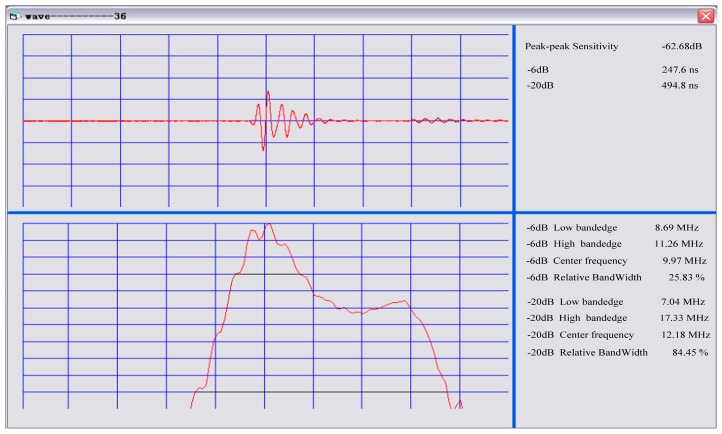
Pulse-echo testing.

First, an pules-echo test was performed on a single array element, and Fourier transform was performed for the convenience of analysis, as shown in Figure 13. As can be seen from the frequency domain, the bandwidth was narrow. We believe that the pressure-welded wire needs to be filled with pure glue to protect it instead of directly filling the matching layer, so the waveform was not ideal, and we can improve it in later experiments.

**Figure 14:**
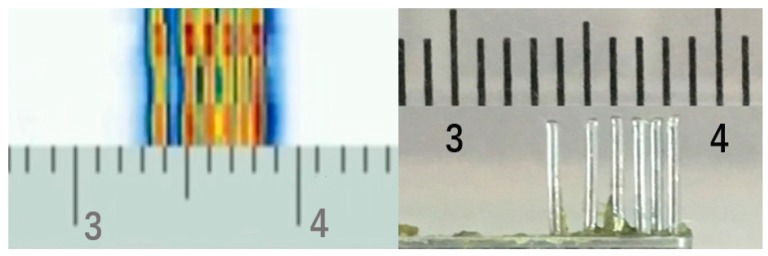
C-scan pulse-echo imaging of a homemade mold.

Water-immersed imaging experiments were conducted using a homemade mold as shown in Figure 14. We used 50 V pulses of 50 ns pulse width with various time delays to drive the array elements of the micro-transducer. If all received signals from the pMUTs are collected and processed, the received beam-forming could be used to achieve a higher signal to noise ratio (SNR) and imaging resolution [21]. Since the micro-transducer array is only 5.12 mm × 3 mm, it is too small to fully image features larger than its own size using electronic scanning. Transmit beam-forming was used to obtain a narrow beam focused 2 mm away from the array, and the steel platform was scanned mechanically at 1000 μm/s. Figure 14 shows the C-scan image of the homemade mold. The mold with different spacing and dimensions of approximately 400 μm was clearly imaged with good image contrast. Considering the limitations of the instrumentation for echo signal processing, the axis imaging effect was not ideal, and the equipment for collecting and processing the echo signals will be improved later to better analyze echo imaging performance.

## 5. Conclusions

This work innovatively designed a microscale linear phased-array ultrasonic transducer based on PZT and elaborated on its structure design and preparation technology. The lateral sound field was analyzed theoretically and verified by experiments. Due to the influence of the hydrophone performance and experimental environment, the transverse sound field experimental effect was not ideal, and the size of the hydrophone and experimental environment will be further improved in the in further experiments. In the pulsed echo imaging experiment, the homemade mold with a feature size of about 400 μm had a good imaging effect with different spacing. In conclusion, the transducer designed by this paper is feasible, and the lateral resolution of the pulse echo imaging reached approximately 400 μm.
